# *TRIM63* (MuRF-1) gene polymorphism is associated with biomarkers of exercise-induced muscle damage

**DOI:** 10.1152/physiolgenomics.00103.2017

**Published:** 2017-12-06

**Authors:** P. Baumert, G-REX Consortium,, M. J. Lake, B. Drust, C. E. Stewart, R. M. Erskine

**Affiliations:** ^1^Research Institute for Sport & Exercise Sciences, Liverpool John Moores University, Liverpool, United Kingdom; ^2^Institute of Sport, Exercise & Health, University College London, London, United Kingdom

**Keywords:** delayed-onset muscle soreness (DOMS) phenotype, E3 ubiquitin-protein ligase, exercise-induced muscle damage (EIMD), MURF1 gene, single nucleotide polymorphism (SNP), tripartite motif containing 63 (TRIM63)

## Abstract

Unaccustomed strenuous exercise can lead to muscle strength loss, inflammation and delayed-onset muscle soreness, which may be influenced by genetic variation. We investigated if a missense single nucleotide polymorphism (A>G, rs2275950) within the *TRIM63* gene (encoding MuRF-1 and potentially affecting titin mechanical properties) was associated with the variable response to unaccustomed eccentric exercise. Sixty-five untrained, healthy participants (genotyped for rs2275950: AA, AG, and GG) performed 120 maximal eccentric knee extensions (ECC) to induce muscle damage. Isometric and isokinetic maximal voluntary knee extension contractions (MVCs) and muscle soreness were assessed before, immediately after, and 48 h after ECC. AA homozygotes were consistently stronger [baseline isometric MVC: 3.23 ± 0.92 Nm/kg (AA) vs. 2.09 ± 0.67 Nm/kg (GG); *P* = 0.006] and demonstrated less muscle soreness over time (*P* = 0.022) compared with GG homozygotes. This may be explained by greater titin stiffness in AA homozygotes, leading to intrinsically stronger muscle fibers that are more resistant to eccentric damaging contractions.

## BACKGROUND/MOTIVATION FOR THE STUDY

Unaccustomed strenuous exercise can lead to muscle strength loss, inflammation, and delayed-onset muscle soreness, which are biomarkers of exercise-induced muscle damage (EIMD) (1). Insufficient recovery from EIMD may increase injury risk, and there is evidence that people recover at different rates, which may be influenced by genetic variation (1).

Muscle RING finger-1 (MuRF-1) is known to be associated with cardiomyopathy and muscle atrophy (5), and it plays a crucial role in the ubiquitin proteasome system by attaching ubiquitin polymers to damaged myofibrillar proteins following EIMD, thus enabling the 26S-proteasome complex to degrade these ubiquitin-marked proteins (3). MuRF-1 is localized at the Z- and also the M-line of the sarcomere, where it also interacts with the strain-sensing kinase domain of the sarcomeric protein, titin (2). This kinase domain is thought to modulate the mechanical properties of titin and the gene expression of other muscle-encoding proteins (4).

MuRF-1 is encoded by the human tripartite motif containing 63 (*TRIM63*) gene, in which a missense single nucleotide polymorphism (SNP, A>G, rs2275950) at amino acid 237 causes a change from lysine to glutamate. This may decrease MuRF-1’s affinity for the titin strain-sensing kinase domain, thus reducing muscle fiber stiffness and increasing its susceptibility to EIMD. To our knowledge, however, this SNP has not yet been linked to any phenotypic trait. We therefore aimed to investigate an association between this SNP and EIMD in untrained young men and women. We hypothesized that *1*) the minor G-allele would be associated with lower muscle strength, and *2*) G-allele carriers would exhibit a greater damage response following maximal eccentric exercise compared with AA homozygotes.

## PHENOTYPE

To induce quadriceps femoris muscle damage, familiarized participants performed 12 sets of 10 maximal eccentric unilateral contractions of the knee extensors. Maximum isometric (at 80° knee flexion; 0° = full extension) and isokinetic (60°/s) voluntary knee-extension contraction (MVC) torque (Humac Norm, CSI, Stoughton, MA, and Biodex Multi-Joint System 3 Pro, Shirley, NY) normalized to body mass, muscle soreness (measured by visual analogue scale in conjunction with a three repetition bodyweight squat) were assessed before, immediately after, and 48 h after the EIMD intervention. Venous blood samples were collected from an antecubital vein for genotypic (1 × 10 ml; BD EDTA vacutainer) and serum analysis (3 × 10 ml; BD serum collection vacutainer). Serum tubes were centrifuged at 1,300 *g* for 15 min (4°C), and all samples were stored at −80°C. Serum creatine kinase (CK) activity (Catachem, Oxford, CT) was analyzed according to manufacturer protocols.

### 

#### Cohort details.

Following ethical approval from Liverpool John Moores University Ethics Committee, and in accordance with the Declaration of Helsinki, written informed consent was obtained from our population cohort, which comprised young, untrained, healthy (identified by physical activity and health questionnaires) female (*n* = 39) and male (*n* = 26) Caucasians (mean ± SD: age = 22.5 ± 4.0 yr; height = 1.71 ± 0.09 m; body mass = 70.9 ± 14.4 kg). Inclusion criteria were: aged between 18 and 35 yr and no history of *1*) leg strength training in the past 6 mo, *2*) muscle-tendon injury in the last 12 mo, and *3*) bone fracture in the lower limbs. Participants were requested to maintain their normal daily routine and to refrain from consuming alcohol or participating in any exercise for 2 days before the study and throughout the intervention.

#### Type of study.

For this candidate SNP study, extraction of the DNA was carried out using QIAamp DNA Blood Kit (Qiagen, Crawley, UK) and following the spin protocol for DNA purification from whole blood. Real-time polymerase chain reaction (PCR) was performed with Rotor-Gene Q (Qiagen) to determine *TRIM63* (A>G, rs2275950) genotype in each participant. Reactions were completed on a 72-well rotor-disk. Each 10 µl reaction volume contained: 5 µl Genotyping Master Mix, 0.5 µl genotyping assay mix (Applied Biosystems, Foster City, CA), 3.5 µl nuclease-free H_2_O (Qiagen), and 1 µl DNA. For control wells, 1 µl nuclease-free H_2_O replaced the DNA template. The following PCR protocol was used: 40 cycles of incubation at 92°C for 15 s (denaturation), then annealing and extension at 60°C for 1 min. Lastly, genotype was determined in all samples (analyzed in duplicate), using Rotor-Gene Q Software 2.3.1 (Qiagen).

#### Details of the SNP studied.

The investigated SNP (rs2275950) is located in the Tripartite Motif Containing 63 gene (*TRIM63*), on chromosome 1 at position 26,058,512 (dbSNP Build 150). This missense A>G (adenine to guanine) SNP has not previously been associated with the response to exercise. The frequency of the effect (major) A allele is 0.77. Linkage disequilibrium (LD) calculations for the *TRIM63* SNP were performed using the LDlink suite and data from the 1000 Genomes Project European ancestry populations (for references, see Appendix 3). (The online version of this article contains supplemental material.)

#### Analysis model.

Data for each parameter were assessed for normal distribution with the Shapiro-Wilk test and by inspection of the Q-Q plots. Hardy-Weinberg equilibrium was determined for the *TRIM63* SNP using a χ^2^ test. Two-way mixed ANCOVAs [within-subjects factor: time (pre, post, and 48 h post; between-subjects factor: genotype (AA, AG, GG); covariate: sex] with Tukey post hoc tests were used to detect associations between the *TRIM63* SNP and isometric and isokinetic MVC knee-extension torque (normalized to body mass), muscle soreness, and serum CK activity over time. All data are presented as means (± SD), and statistical significance was identified when *P* < 0.05. All MVC data were recorded in AcqKnowledge software (version 4.2; Biopac-Systems, Goleta, CA), and statistical analysis was performed with SPSS (v23; IBM, Armonk, NY).

## RESULTS

Genotype frequency distribution for the *TRIM63* (rs2275950; X^2^ = 1.156, *P* = 0.282) SNP was in Hardy-Weinberg equilibrium. Forty participants were AA, 20 were AG, and five were GG genotype. Normalized isometric (*P* < 0.001) and isokinetic (*P* < 0.001) MVC, leg muscle soreness (*P* < 0.001), and CK activity (*P* = 0.01) all showed a main effect of time, indicating EIMD. There was a main effect for genotype regarding normalized isometric (*P* = 0.006, Fig. 1) and isokinetic (*P* = 0.031) MVC, with AA stronger than GG in both cases [isometric MVC normalized to body mass at pre-EIMD: 3.23 ± 0.92 Nm/kg (AA) vs. 2.67 ± 1.09 Nm/kg (AG) vs. 2.09 ± 0.67 Nm/kg (GG)]. For figures, see online supplemental material. There was a genotype × time interaction regarding muscle soreness (*P* = 0.022), with AA homozygotes showing attenuated muscle soreness 48 h post-EIMD compared with AG and GG genotypes (2.9 ± 2.2 cm vs. 4.5 ± 2.3 cm and 5.4 ± 2.2 cm, respectively; Fig. 2).

## INTERPRETATION

Homozygotes of the major *TRIM63* A-allele were stronger (relative to body mass) and recovered more quickly following strenuous exercise compared with G-allele carriers. It is possible that the A-allele is linked to a higher affinity for titin’s strain-sensing kinase domain, leading to greater kinase activation. This may improve the mechanical properties of titin, enabling a stiffer muscle fiber to transmit more force laterally and longitudinally, potentially making the fiber intrinsically stronger and more resistant to acute damaging eccentric contractions. However, rs2275950 is in LD with other *TRIM63* SNPs, and with SNPs within the exostosin-like 1 (*EXTL1*) and the solute carrier family 30 member 2 (*SLC30A2*) genes. Nevertheless, given MuRF-1’s role in muscle protein degradation and with titin’s strain-sensing kinase domain, rs2275950 is a likely genetic determinant of the interindividual variability in the human response to EIMD. Further work is necessary to elucidate if *1*) other *TRIM63*, *EXTL1*, and/or *SLC30A2* SNPs (either in combination or isolation) are of functional significance regarding EIMD; *2*) this quicker recovery following an acute bout of intense exercise in AA homozygotes compared with G-allele carriers translates into greater muscle hypertrophy and/or strength gains following chronic resistance training.

## DISCLOSURES

No conflicts of interest, financial or otherwise, are declared by the authors.

## AUTHOR CONTRIBUTIONS

P.B., B.A., J.A.C., V.E., and K.O.J. performed experiments; P.B. and R.M.E. analyzed data; P.B., C.S., and R.M.E. interpreted results of experiments; P.B. prepared figures; P.B. drafted manuscript; P.B., M.J.L., B.D., C.S., and R.M.E. edited and revised manuscript; P.B., M.J.L., B.D., C.S., and R.M.E. approved final version of manuscript; R.M.E. conceived and designed research.

## Supplemental Data

Appendix 1 Appendix 1 - .pdf (160 KB)

Appendix 3 Appendix 3: List of the G-REX (Genetics, Recovery and EXercise) consortium members and additional references - .docx (15 KB)

Table 1 Table 1 - .xlsx (12 KB)

Table 2 Table 2 - .xlsx (13.7 KB)
