# Supplementary material for: TRIM63 (MuRF-1) gene polymorphism is associated with biomarkers of exercise-induced muscle damage
Source: Physiol Genomics. 2017 Dec 6;50(3):142–3. doi: 10.1152/physiolgenomics.00103.2017 (PMC5899231; doi:10.1152/physiolgenomics.00103.2017)
Supplement: Appendix 1 — pdf (160 KB) [file appendix1.pdf]

Appendix 1_1

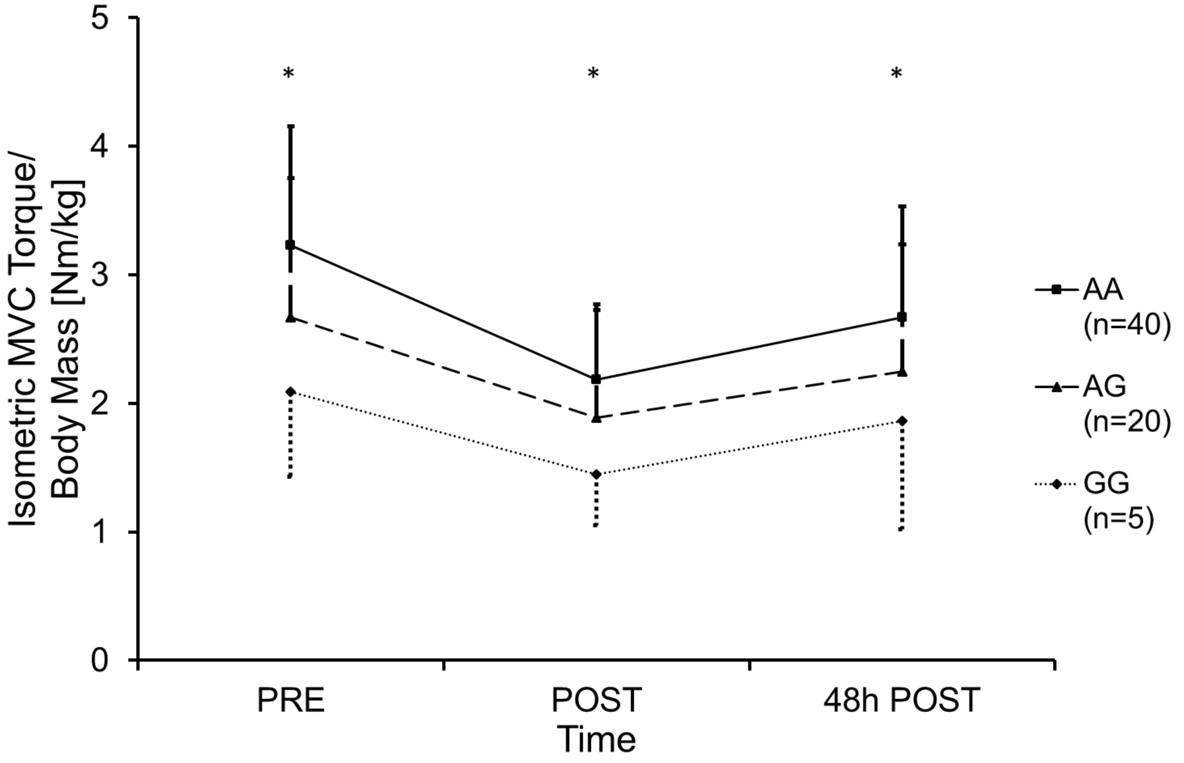

*Figure 1. Isometric knee extension maximal voluntary contraction torque normalized to body mass for individuals of the three TRIM63 (A>G, rs2275950) genotypes before (Pre), directly after (Post) and 48h after (48 h Post) a strenuous exercise intervention. * indicates significant differences ($p < 0.05$) between the AA and GG genotypes. MVC = Maximal voluntary contraction. Values are mean ± SD.*

Appendix 1_2

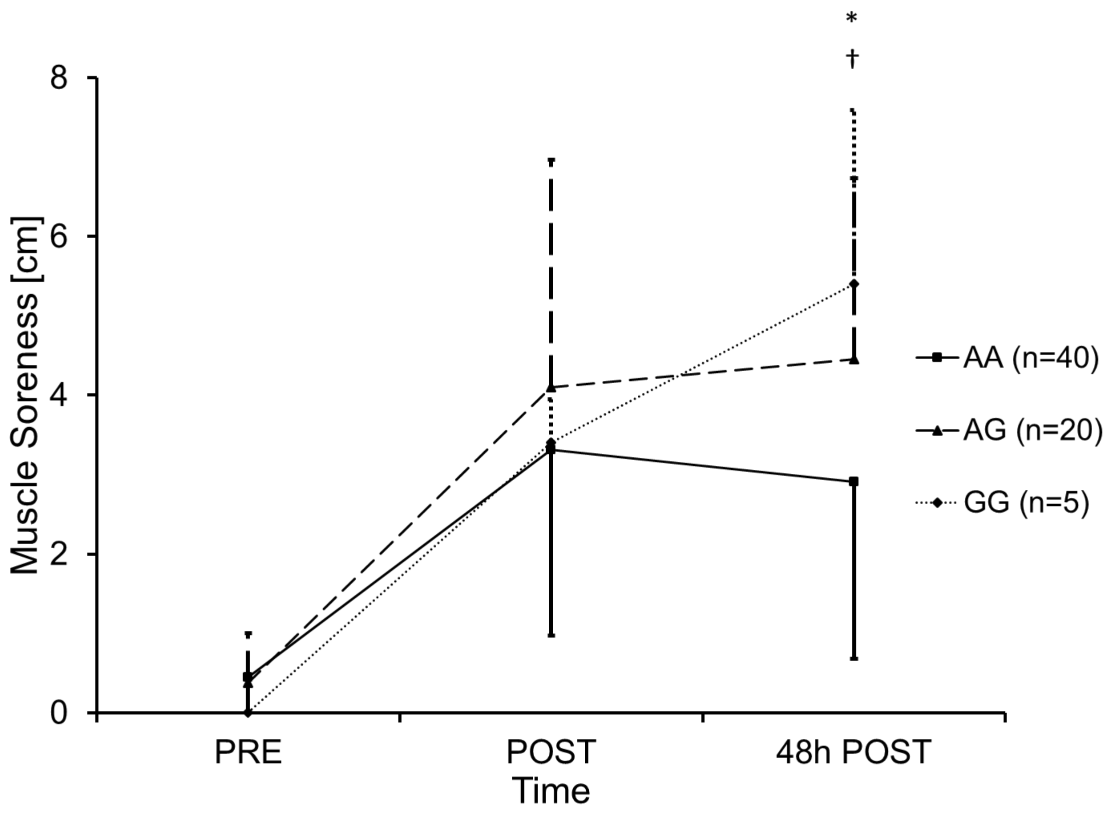

*Figure 2. Muscle Soreness measured via Visual Analogue Scale for individuals of the three TRIM63 (A>G, rs2275950) genotypes assessed before (Pre), directly after (Post) and 48h after (48 h Post) 120 a strenuous exercise intervention. * indicates significant differences (p< 0.05) between AA and GG genotypes. † indicates significant differences (p< 0.05) between AA and AG genotypes. Values are mean ± SD.*
